# Tolerance Evaluation of Celery Commercial Cultivars and Genetic Variability of *Fusarium oxysporum* f. sp. *apii*

**DOI:** 10.3390/microorganisms11112732

**Published:** 2023-11-09

**Authors:** Mónica Blanco-Meneses, Mauricio Serrano-Porras, Anny Calderón-Abarca, Alejandro Sebiani-Calvo, Gabriel Vargas, Oscar Castro-Zúñiga

**Affiliations:** 1 Plant Protection Research Center (CIPROC), Molecular Biology Department, Agronomy School, Universidad de Costa Rica, San Jose 11801, Costa Rica; anny.calderon@ucr.ac.cr (A.C.-A.); alejandro.sebiani@ucr.ac.cr (A.S.-C.); juangabriel.vargas@ucr.ac.cr (G.V.); 2 Plant Protection Research Center (CIPROC), Phytopathology Department, Agronomy School, Universidad de Costa Rica, San Jose 11801, Costa Rica; mauricio.serrano@ucr.ac.cr (M.S.-P.); oscar.castrozuniga@ucr.ac.cr (O.C.-Z.)

**Keywords:** horticulture, greenhouse crops, field crops, *Apium graveolens*, *Fusarium* yellows, Foa race 3, molecular variability

## Abstract

Celery (*Apium graveolens* var. *dulce)* is affected by several plant diseases, such as *Fusarium oxysporum* f. sp. *apii* (Foa). Four Foa races have been found in the US. The goals of this study were to determine which races are present in Costa Rica and to quantify the tolerance of the imported commercial cultivars of celery produced in the country. Isolates from 125 symptomatic celery plants from three different geographical locations were analyzed, 65 of which were selected for phylogenetic analysis. All isolates presented a short sequence of five nucleotides that differentiates Foa race 3 in the IGS rDNA region. Three different haplotypes closely related to race 3 were found, which were highly virulent, produced great losses, and affected all cultivars (resistant to races 2 and 4) of imported commercial celery. Additionally, five different cultivars of celery were evaluated against seven pathogen isolates identified as race 3 in greenhouse conditions. Two of the cultivars showed significantly less chlorosis, wilting, mortality, and higher fresh weight. Most of the Foa isolates significantly increased chlorosis, wilting, and mortality compared to non-inoculated control. Celery producers in Costa Rica lack access to seeds resistant to the Foa race 3 present in the country.

## 1. Introduction

Celery (*Apium graveolens* var. *dulce*) is a member of the Umbelliferae or Apiaceae family. Other vegetables in this family include carrots, parsley, and parsnip. This plant was domesticated in the 17th century and is native to areas surrounding the Mediterranean. It comes from wild *Apium graveolens* that grows in the mountain regions of Southeast Asia and swampy areas of North Africa and Europe [1,2]. Celery is cultivated worldwide and is an excellent source of vitamins, phenolic compounds, and other nutrients [3]. It is widely cultivated for its fleshy leafstalk used as a vegetable and seeds that yield essential oils [3,4].

The United States, Spain, Mexico, and China are the world’s major celery producers. In 2018, the United States of America (US) produced (94% in California) close to 800,000 metric tons on 30,000 acres, representing an income of $421 million (US dollars) to the celery market [5]. Commercialization of seeds is common in some countries, such as China, India, some European countries, and the US. Indian celery seeds dominate the world market, with most seeds exported to Europe and America [4,6].

Celery is affected by several plant diseases, both in the field and in the postharvest stages, including downy mildew (*Plasmopara nivea* Schr.), early blight (*Cercospora apii* Fresen.), septoria or late blight (*Septoria apiicola* Speg.), soft stem rot (*Sclerotinia sclerotiorum* Lib. De Bary and *S. minor*), and soft rot and stem rot caused by *Pectobacterium carotovorum* subsp. *carotovorum* [7,8,9]. In the last decade, the wilt caused by *Fusarium oxysporum* f. sp. *apii* (Foa) has taken importance with symptoms including growth retardation, stunting, dwarfing, yellowing of the foliage, foliage wilting, root system damage (with orange-brown coloration in the vascular tissue in some advanced stages of the disease), and appearance of soft rot on the crown [10,11].

The *Fusarium oxysporum* species complex (FOSC) contains thousands of clonal lineages, with individual strains typically causing disease in a limited number of plant hosts. Plant host specificity led to the *forma specialis* (f. sp.) designation; for example, in FOSC f. sp. *apii* (Foa), the pathogen only causes disease in celery (*Apium graveolens* var. *dulce*) [12,13]. In celery, four pathogenic Foa races are known and have been described in the US according to the cultivars. Race 1 responds to the pathogen that only affects yellow celery, among which “Golden Boy”, is one of the best known. In 1952, a new variety of celery with high resistance to Foa was introduced to the US market, and the prevalence of this race was reduced. A new race appeared in the 1970s that affected both green and yellow cultivars and was identified as race 2 [14]. Race 3 is virulent only to green celery. It has been less frequent since its last appearance in 1984, and it is currently believed to pose no risk [15]. On the other hand, race 4 was described in 2013 in California and was reported as the most aggressive race, infecting both green (all cultivars) and yellow celery [16]. It is common at temperatures above 22 °C [5] and has been spreading within California since its discovery. Currently, race 4 cannot be controlled via either host resistance or chemical methods that reduce the pathogen abundance in infested soils [17].

Some new cultivars with high tolerance to Foa have been introduced to the celery market in the US, including Challenger, which is resistant to races 2 and 4 [18]. The resulting commercial celery cultivars, such as Command, Green Bay, Tall Utah 52–70 R Improved, and Sabroso, have been the major tools for *Fusarium* yellows management since the early 2000s [13].

More recently, Epstein et al. [16] performed virulence tests and a two-gene dataset for isolates collected between 1993 and 2013. They found a unique and highly clonal population of Foa race 2 before 2013. After 2013, new highly virulent clonal isolates, designated as race 4, were discovered. They found that races 1, 3, and 4 are similar in sequence but have significant differences in virulence, allowing the pathogen to evolve into new races.

*Apium graveolens* var. *dulce* green color is the only variety used in Costa Rica. The origins of the introduction of the pathogen *Fusarium oxysporum* f.sp. *apii* in Costa Rica is unknown. The current hypothesis is that the pathogen was introduced through seed material, as there is no seed production in the country. Foa causes aggressive symptoms and losses on field and greenhouse celery. The predominant Foa race (s), as well as how resistant are the celery cultivars to these Foa races in the country, are unknown.

The objective of this study was to determine which races identified by Epstein [16] are present in celery in Costa Rica. We sequenced two genetic regions to perform a phylogenetic analysis and quantify the tolerance of the imported commercial cultivars of celery to Foa isolates from different geographical locations.

## 2. Materials and Methods

### 2.1. Collection and Isolation of Fusarium spp.

**Figure 1:**
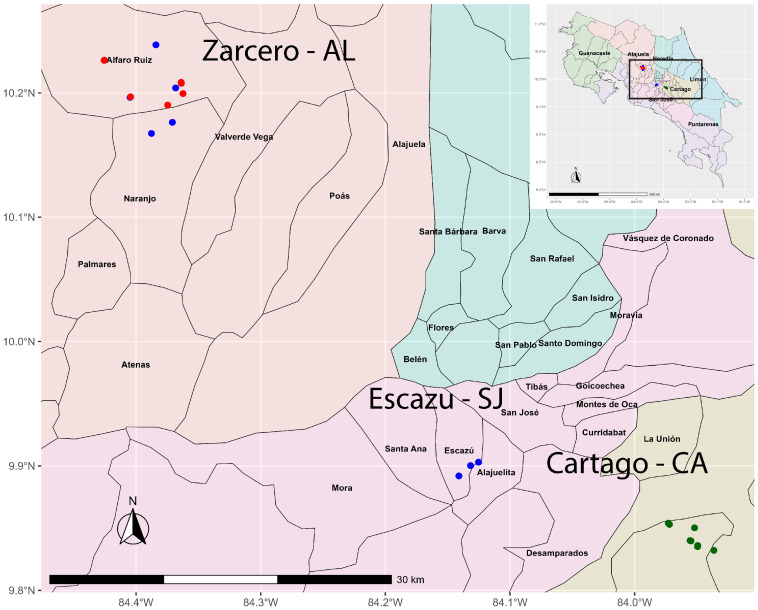
Collected symptomatic celery samples for Foa. On the map, three different locations: Zarcero-Alajuela, Escazú-San José, and Cartago, in Costa Rica. Locations in green, blue, and red were sampled in 2018, 2019, and 2020, respectively.

From 2018 to 2020, one hundred twenty-five diseased celery plants (symptomatic for *Fusarium* yellow) were collected from different geographical locations (Figure 1). Plants were collected from open fields (Cartago-CA and Escazu-SJ) and greenhouses (Zarcero-AL). The three locations presented differences in elevation and climate conditions: Cartago’s elevation is 2.270 m a.s.l. and temperatures range from 11–18 °C; Escazú’s elevation is 1.200 m a.s.l. and temperatures range from 20–26 °C; Zarcero’s elevation is 1.736 m a.s.l., with temperatures ranges from 12–22 °C. In Escazú and Cartago, celery is grown in open fields, while it is grown predominately in greenhouses in Zarcero.

Small segments of 1 cm^2^ from on the crown and root tissue of celery plants were surface-disinfected by immersion in 1% sodium hypochlorite solution for 1 min, followed by 70% ethanol for 1 min. Each sample was rinsed three times with sterile distilled water. A quarter-strength potato dextrose agar (¼ PDA) (Oxoid CM0139, Thermo Fisher Scientific, Waltham, MA, USA) was autoclaved at 121 °C for 20 min and acidified with 0.075% *v*/*v*, 50% lactic acid. Five pieces of 3 × 1.5 mm from the advancing margin of the lesions were cut and plated onto ¼ PDA. The plates were incubated for 7 days at 24–25 °C. The colonies with appearance and morphology consistent with *Fusarium* were transferred into a new ¼ PDA plate and incubated for another 14 days. A spore suspension of 1 × 10^2^ in an autoclaved water agar media (Oxoid LP0011, Thermo Fisher Scientific, Waltham, MA, USA) was used to make monosporic cultures, which were incubated at 24–25 °C for 1 day. One spore from each culture was then sub-cultured in acidified ¼ PDA and incubated for 7 days. The axenic culture was stored by taking 0.5 cm^2^ of mycelia from a monosporic culture grown on sterile filter paper and stored at −80 °C. Eighty-two isolates of Foa were selected for analysis.

### 2.2. DNA Extraction and Procedures

Total genomic DNA was extracted from 0.5 mg of mycelia obtained from seven-day-old *Fusarium oxysporum* f.sp. *apii* monosporic, cultured on ¼ PDA using the standard cetyltrimethylammonium bromide (CTAB) protocol [19]. The DNA pellet was dissolved in 50 μL of TE buffer. The DNA concentration and purity were determined using a NanoDrop One C spectrophotometer (Thermo Fisher Scientific, Waltham, MA, USA). The following loci were amplified using polymerase chain reaction (PCR): the 700-bp partial CDS of the translation elongation factor 1-alpha (EF-1) gene (TEF-1α) [20] and a fragment of 970-bp including the partial sequence of the 28S ribosomal RNA gene, the complete sequence of the intergenic spacer 28S-18S ribosomal RNA and the partial sequence of the 18S ribosomal RNA gene (IGS rDNA) [16,21] (Table S1). The PCR reaction was performed in a 25 μL mixture with 0.25 μL DreamTaq DNA Polymerase [5 U/μL] (Thermo Sientific, Waltham, MA, USA, Ref. EP0702), 2.5 μL 10× Dream Taq Buffer (included [20 mM] MgCl_2_, Thermo Scientific), 2.5 μL dNTP Mix [each 2 mM] (Thermo Scientific Ref. R0241), 1.25 μL of each forward and reverse primers [10 nM], 1 μL of the template DNA [10–30 ng] and 16.25 μL sterile ultrapure water. PCR program consisted of an initial denaturing step of 95 °C for 120 s, followed by 35 cycles of 96 °C for 60 s, 52 °C (TEF-1α) and 57 °C (IGS) for 60 s, and 72 °C for 60 s, and a final extension at 72 °C for 10 min on a Mastercycler Pro Thermal (Eppendorf, Hamburg, Germany).

The amplified products were purified with the Exonuclease I (ExoSapI) (Thermo Scientific, Waltham, MA, USA, ref. EN0582) and sequenced at Macrogen Inc., Gangnam-gu, Seoul, Republic of Korea. All sequences were manually checked for quality, trimmed, and aligned with either ClustalW2 or Muscle. All polymorphisms were checked manually. NCBI GenBank accession numbers for genetic variable isolates are OR640020 to OR640041 for the TEF-1α and OR626669 to OR626690 for IGS rDNA.

### 2.3. Phylogenetic Analysis

Phylogenetic analysis utilized the concatenated TEF1 and IGS rDNA sequences from 65 isolates. The study also included sequences from Foa races 1, 2, 3, and 4 [16,21] and the sequence of *F. foetens*, which was selected as the outgroup for the phylogeny [16]. Identical sequences were collapsed to minimize the redundancy in the analysis. The concatenated sequences were aligned using the MAFFT algorithm as implemented on the GUIDANCE2 server [22] (available at: http://guidance.tau.ac.il, accessed on 16 May 2023) with 1000 bootstrap replicates. The concatenated alignment was 1.3 kb. The quality of the alignment was also evaluated using GUIDANCE2. The best evolutionary model was determined with JModeltest v2.1.9 [23]. The phylogenetic trees were constructed using the maximum likelihood method implemented in PhyML3.1 [24] with the “General Time Reversible” (GTR + G) model and with 1000 bootstrap replicates. Finally, the trees were edited in iTOL [25] (available at: https://itol.embl.de/, accessed on 16 May 2023).

### 2.4. Disease Tolerance Evaluation

#### 2.4.1. Celery Cultivars 

Five celery cultivars commercially grown in Costa Rica were evaluated. The Merengo cultivar and the IseI were collected at each sampled location (Cartago, Escazu, and Zarcero). The David cultivar was collected in Cartago, the Gigante Verde cultivar was only collected in Zarcero, and the Triumph cultivar only in Escazu. Isel cultivar was the newest material introduced in the country with unknown resistance or susceptibility to Foa.

Four-week-old seedlings commercially produced by importer seed suppliers nationwide were used in this experiment.

#### 2.4.2. Pathogen Isolates and Inoculum Production

Seven pathogenic isolates from three geographically separated areas were used for the disease tolerance experiment. They were selected according to molecular identification and phylogenetic analysis. Each isolate was grown for 14 days on ¼ PDA for this experiment. The inoculum was prepared in autoclavable gas exchange filter bags. Autoclavable bags with a filter patch (Myco Supply Co. Inc., Pittsburg, PA, USA) were filled with 400 g of rice grains and autoclaved twice for 20 min, with 24 h between each cycle. After cooling down overnight, they were inoculated with 1.5 plates with the cultured fungus and cut into pieces of approximately 0.5 cm^2^. The inoculated bags were kept in the dark for 14 days in an incubator (Thermo Scientific, Waltham, MA, USA) at 24–25 °C. The bags were gently shaken once per day to prevent clumps.

#### 2.4.3. Greenhouse Conditions

This trial was carried out in a greenhouse at the Plant Protection Research Center (CIPROC) at the University of Costa Rica from August to November 2021. Temperatures ranged from 20–25 °C. Polyethylene pots (532 mL) were used with three 2 mm diameter holes to allow excess water to drain. The pots were filled with a 1:1 mixture of soil and peat moss (90%) and fine perlite (10%) (Jiffy Products of America Inc., Lorain, OH, USA). Vessels were filled with 250 mL of the soil-peat mixture, a layer of 5 g of inoculum, and another layer of 250 mL of the soil-peat mixture. Subsequently, 4-week-old seedlings were sown. The plants were irrigated daily with 100 mL of water.

#### 2.4.4. Experimental Design and Data Analysis

The experimental design was completely randomized with a 5 × 8 factorial treatment arrangement (5 cultivars of celery and 7 isolates of the pathogen plus a control) and 4 replicates with 5 observational units per replicate (pot). Each experimental unit consisted of 5 pots. Six weeks after the transplant, the following variables were evaluated: the total number of leaves, yellowing, the occurrence of wilting in the plant, death of the plant, plant fresh weight, and root fresh weight. The data was analyzed using R software version 4.0.4 and RStudio version 2022.02.03 using agricolae and car R packages. An analysis of variance was performed in a linear model with Variety and Isolation of the pathogen as fixed factors and the interaction of Variety*Isolation. A Tukey mean comparison (alpha = 0.05) was performed for the combination of treatments. The Levene test (homogeneity of variances) and the Shapiro–Wilks test (normality of residuals) were used to verify the assumptions before the analysis of variance. In cases where the assumptions were not met, data transformation was performed before the analysis of variance. With the data of the percentage of leaves with chlorosis (yellowing), percentage of plants with wilting symptoms, and percentage of mortality, an angular transformation was performed (i.e., the arcsine of the square root of Y was calculated). The means were retransformed to the original units (calculating the squared power of the sine of Y) to present them in the figures.

## 3. Results

A total of one hundred twenty-five plants from different geographical areas in Costa Rica were collected between 2018 and 2020. Eighty-two Foa isolates from the crown and root tissue of celery symptomatic for *Fusarium* were analyzed.

Sequences from TEF1 and IGS rDNA regions were analyzed and compared. Five single nucleotides and one deletion differentiate the Foa race 3 from the other races (Figure S1). The presence of these specific sites was confirmed for 58 sequences from the Costa Rican population.

### 3.1. Phylogenetic Analysis

**Figure 2:**
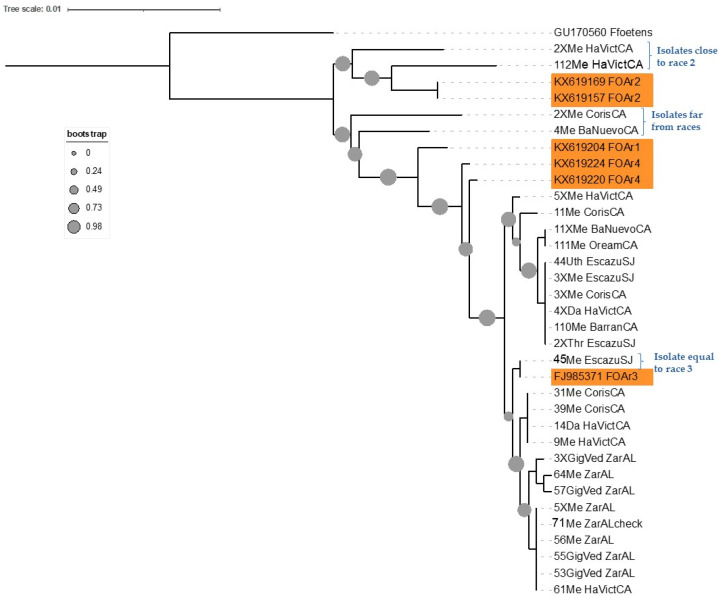
Phylogenetic analysis of *Fusarium oxysporum* f. sp. *apii* from different locations in Costa Rica and compared with US races. Races 1, 2, 3 and 4 are indicated on bold yellow.

The phylogenetic analysis of 65 sequences of two concatenated genes TEF1 and IGS rDNA was performed to compare known US races with Costa Rican isolates. We found high similarity between Costa Rica isolates and the *Fusarium oxysporum* f. sp. *apii* race 3. A total of 54 isolates were closely related to race 3, two variants were placed close to race 2, and three isolates were not related to any race in the tree. There were no isolates that grouped with races 1 and 4 (Figure 2).

**Figure 3:**
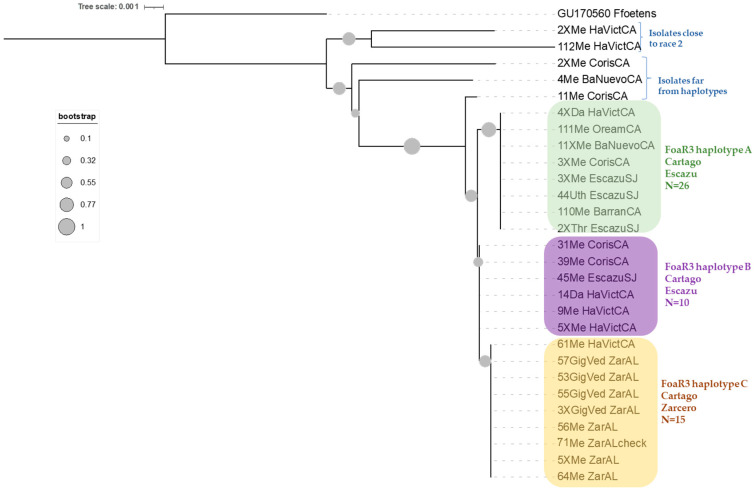
Phylogenetic analysis of different haplotypes of *Fusarium oxysporum* f. sp. *apii* from Costa Rica.

When only Costa Rican sequences were considered (Figure 3), three branches were grouped individually and named race 3 haplotypes A, B, and C (r3hapA, r3hapB, and r3hapC). Haplotype A was formed by 26 sequences from isolates from Cartago and San Jose (frequency CA = 0.77 and SJ = 0.23). These were isolated from four of the celery cultivars used in Costa Rica (freq: David (0.15), Merengo (0.73), Utah (0.04), and Triumph (0.08). Haplotype B was formed by 10 isolates from Cartago and San Jose (freq: CA = 0.9 and SJ = 0.1) isolated from Merengo (freq: 0.9) and David (freq: 0.1) cultivars. The haplotype C was composed of 15 sequences from Zarcero and Cartago (freq: ZA = 0.93 and CA = 0.07) isolated from Merengo (freq: 0.6) and Gigante Verde (freq: 0.4).

### 3.2. Tolerance Evaluation

From the phylogenetic analysis, seven isolates were chosen for the tolerance evaluation. Isolates within race 3 and other closer isolates phylogenetically diverged from the race 3: 11MeCorisCA (mut-α), 28MeCorisCA (mut-β), 110BarrancaCA (r3hapA), 45Escazú (r3hapB), 71Zarcero (r3hapC), and 53Zarcero (r3hapC), and one sequence defined as an isolate closely related to race 2, 112MeHaVictCA (mut-r2). There was a significant effect on the specific cultivar and treatment when the different isolates (similar to Foa race 3, similar to Foa race 2, and other isolates far from the focal races) were placed on different celery cultivars. These effects were observed in the percentage of leaves with yellowing (chlorosis), wilting, mortality, and fresh weight (*p* < 0.0001). In addition, a significant effect on the Cultivar by Treatment interaction was found, for which the effect of the treatment on each cultivar was compared separately. No significant differences were found between cultivars (*p* = 0.4423) for the root fresh weight variable, nor between treatments (*p* = 0.4484). Likewise, the Cultivar*Treatment interaction was not significant (*p* = 0.4933) (Table 1).

**Figure 4:**
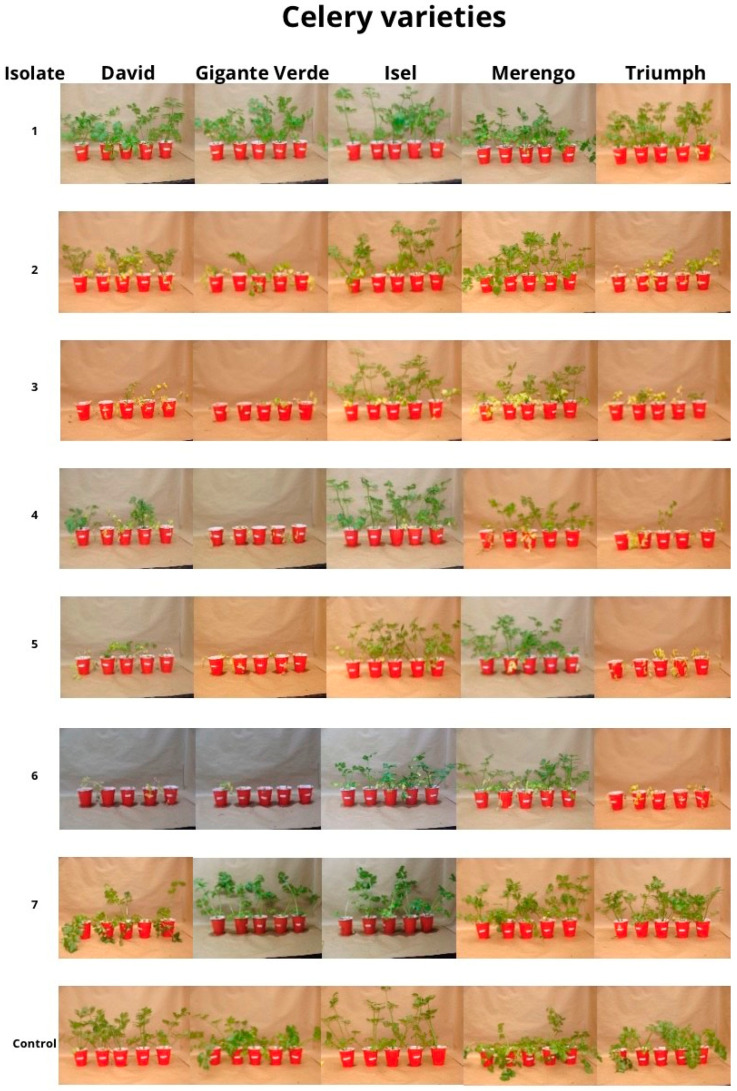
Plant material from five celery cultivars (David, Gigante Verde, Isel, Merengo, and Triumph) inoculated with seven isolates of *Fusarium oxysporum* f. sp. *apii* (1: mut-β, 2: mut-r2, 3: mut-α, 4: r3hapA, 5: r3hapC, 6: r3hapB, and 7: r3hapC) six weeks after plantation.

**Figure 5:**
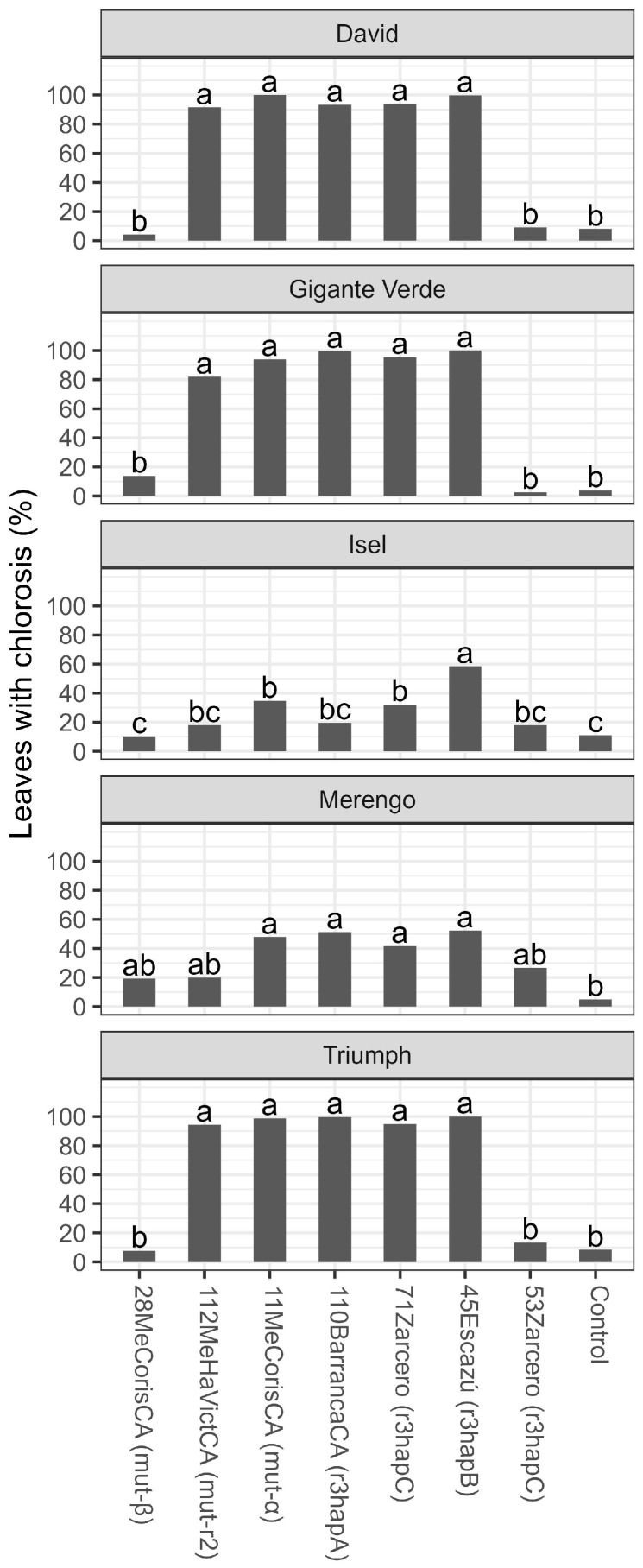
Percentage (%) of leaves with symptoms of yellowing and chlorosis six weeks after transplantation of five celery cultivars inoculated with different isolates of *Fusarium oxysporum* f. sp. *apii* from Costa Rica. The data received angular transformation before the statistical analysis, and the means presented in the figure were retransformed using the squared power of the sine Y. Different letters on the bars indicate significant differences according to the Tukey test (alpha = 0.05).

The inoculation with various isolates of the pathogen produced evident wilting symptoms in the celery plants when compared to the control treatment six weeks after transplanting (Figure 4). The isolates mutr2, mut-α, r3hapA, r3hapB, and r3hapC (71 Zarcero) caused chlorosis symptoms in more than 80% of the leaves in the David, Gigante Verde, and Triumph cultivars. Isolates mut-β, and r3hapC (53Zarcero) were not different from the control treatment for these cultivars. In the Merengo cultivar, the isolates mutr2, mut-α, r3hapA, r3hapB, and r3hapC caused greater chlorosis (no more than 50%) compared to the control. In the Isel cultivar, the isolates caused symptoms of chlorosis of less than 30%, except isolate r3hapB, which exceeded 50% (Figure 5).

**Figure 6:**
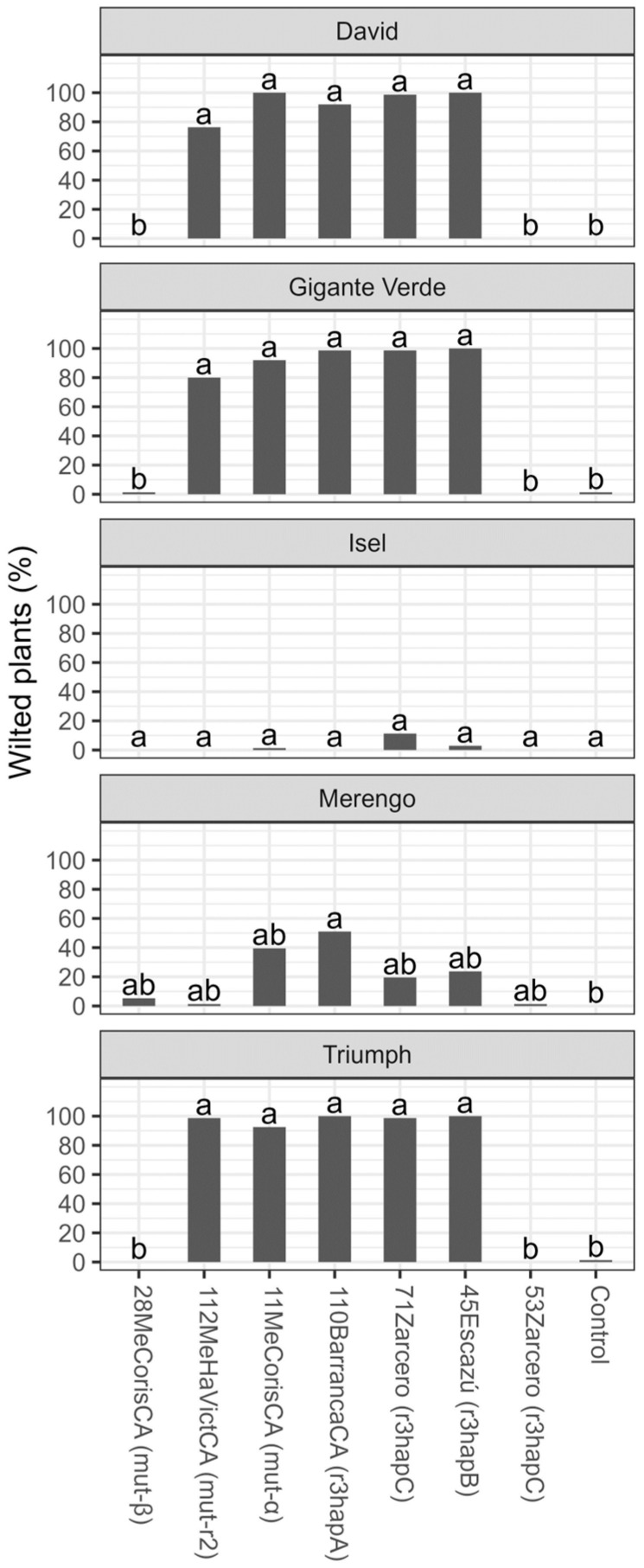
Percentage (%) of plants with wilt symptoms six weeks after transplantation of five celery cultivars inoculated with different isolates of *Fusarium oxysporum* f. sp. *apii* from plantations in Costa Rica. The data received angular transformation before the statistical analysis, and the means presented in the figure were retransformed using the squared power of the sine Y. Different letters on the bars indicate significant differences according to the Tukey test (alpha = 0.05).

Similarly, the isolates mutr2, mut-α, r3hapA, r3hapB, and r3hapC (71Zarcero) caused wilting symptoms in 100% in the David, Gigante Verde, and Triumph cultivars. In the Merengo cultivar, these isolates caused wilting symptoms that did not exceed 50%. In the Isel cultivar, no differences were found concerning the control treatment (Figure 6).

**Figure 7:**
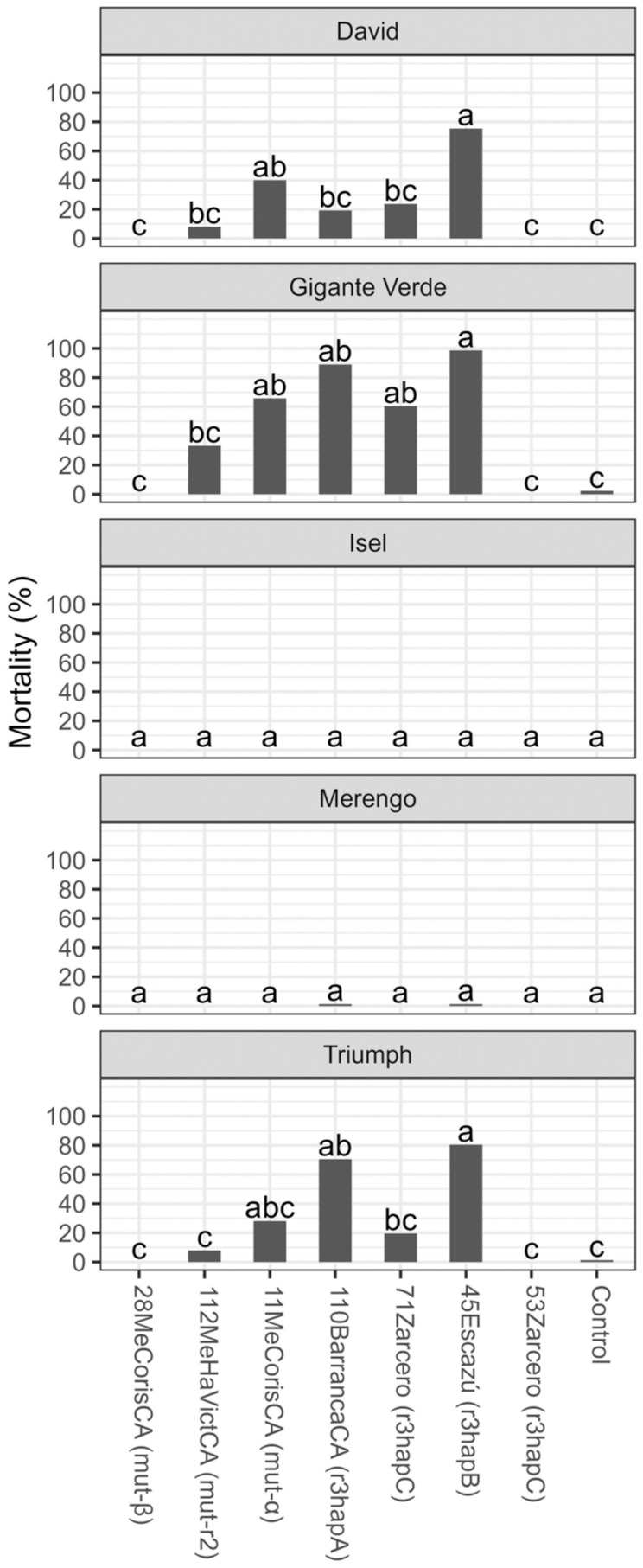
Plant mortality (%) six weeks after transplantation of five celery cultivars inoculated with different isolates of *Fusarium oxysporum* f. sp. *apii* from Costa Rica. The data received angular transformation before the statistical analysis, and the means presented in the figure were retransformed using the squared power of the sine Y. Different letters on the bars indicate significant differences according to the Tukey test (alpha = 0.05).

Consistent with the observations related to chlorosis and wilting, isolates mutr2, mut-α, r3hapA, r3hapB, and r3hapC (71Zarcero) caused greater plant mortality in the David, Gigante Verde, and Triumph cultivars. In particular, the isolate r3hapB caused mortality in more than 70% of plants, while isolates mutr2 and r3hapC were not different from the control treatment in these three cultivars. None of the isolates differed from the control treatment for the Isel and Merengo cultivars (Figure 7).

**Figure 8:**
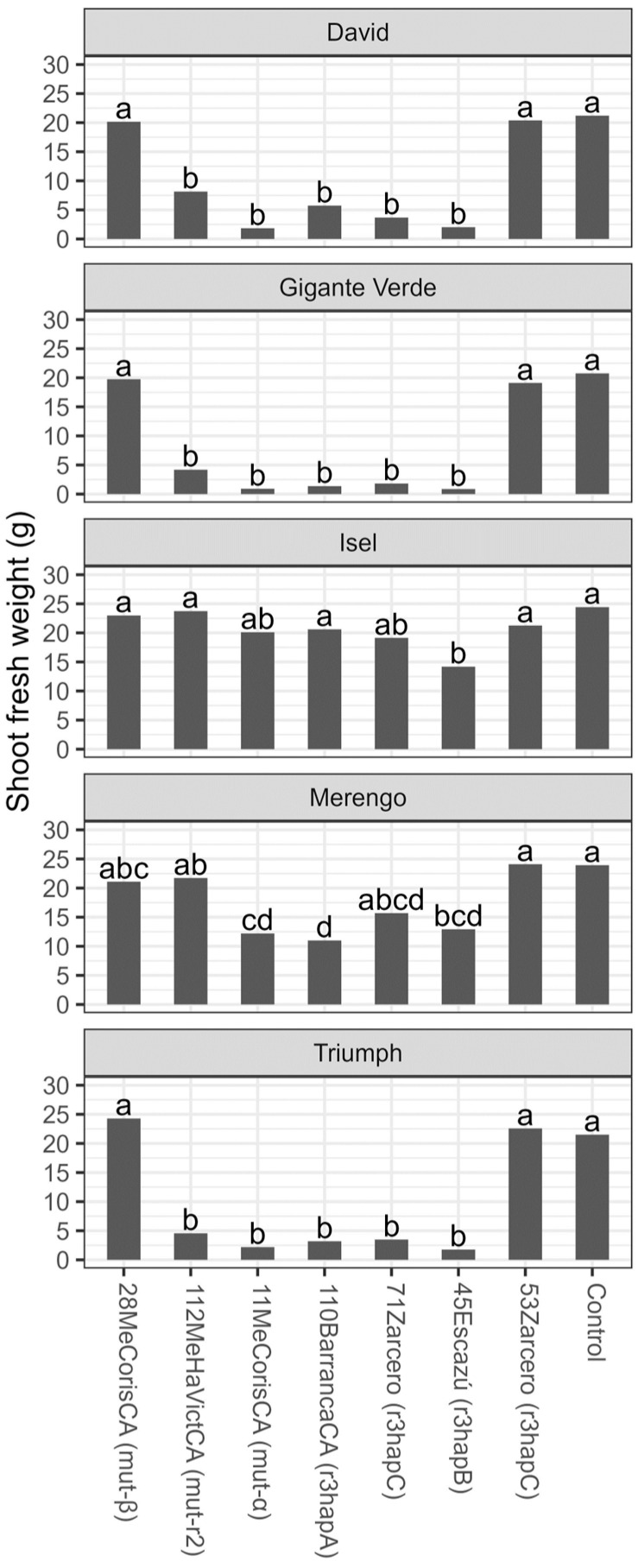
Shoot fresh weight (g) six weeks after transplantation of five celery cultivars inoculated with different isolates of *Fusarium oxysporum* f. sp. *apii* from Costa Rica. Different letters on the bars indicate significant differences according to the Tukey test (alpha = 0.05).

The shoot weight was significantly reduced by the isolates mutr2, mut-α, r3hapA, r3hapB, and r3hapC (71 Zarcero) in the David, Gigante Verde, and Triumph cultivars. In the Isel cultivar, only the r3hapB isolate produced a significant weight reduction, while in the Merengo cultivar, the mut-α, r3hapA, and r3hapB isolates produced a significant weight reduction (Figure 8).

**Figure 9:**
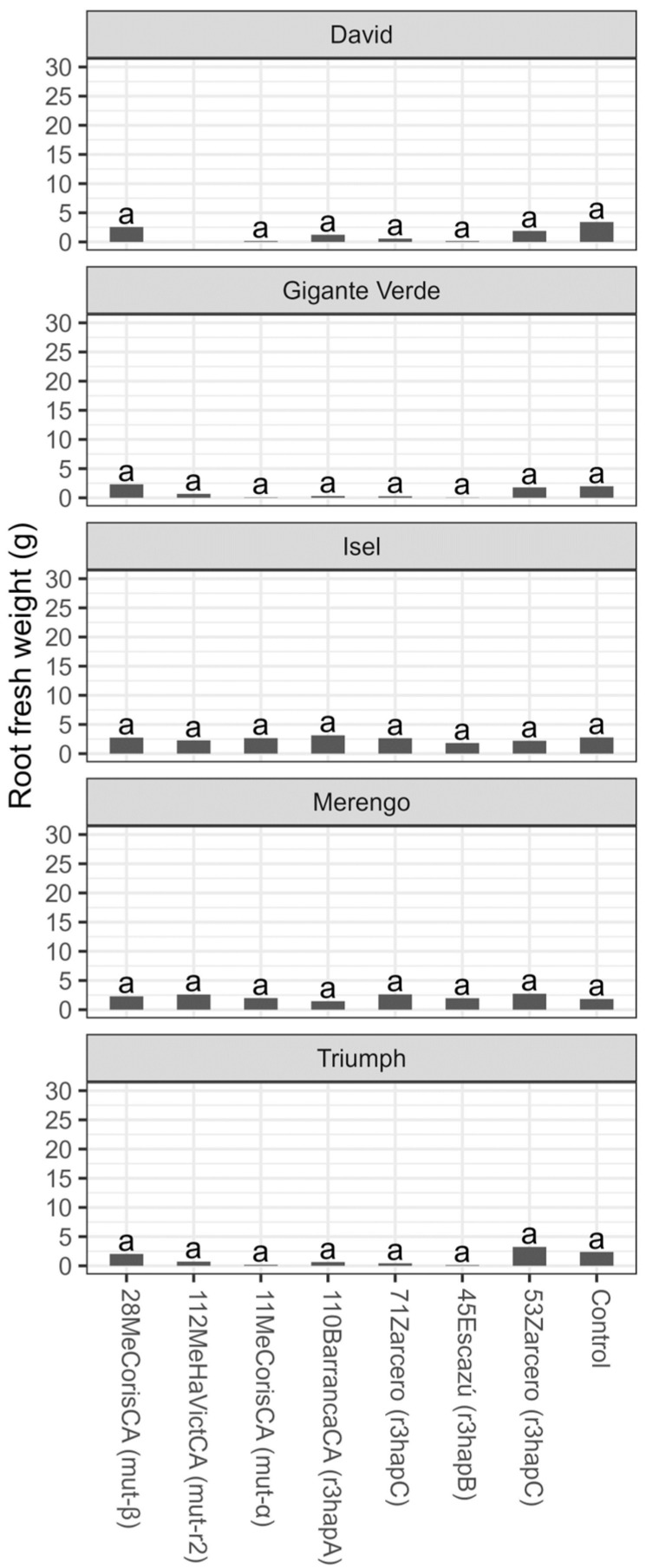
Root fresh weight (g) six weeks after transplanting for five celery cultivars inoculated with different isolates of *Fusarium oxysporum* f. sp. *apii* from plantations in Costa Rica. Same letters on the bars indicate no significant differences according to the Tukey test (alpha = 0.05).

No effect from the Foa isolates was detected on any of the celery cultivars for the fresh weight of the root tissue (Figure 9). The fresh weight of the root did not exceed 4 g in any of the treatments.

## 4. Discussion

Between the years 2015 and 2020, *Fusarium oxysporum* f. sp. *apii* (Foa) was present in all commercial cultivars of *Apium graveolens* var. *dulce* and aggressively affected greenhouse and open-field celery plants in Costa Rica. Differences in elevation or temperature have not affected the development of this pathosystem. Symptoms consist of stunted growth or dwarfing, yellow foliage and brittle, wilting, orange-brown discoloration of root vascular tissue, and, in some cases, spots on petioles and crowns. In the advanced stages of the disease, soft rot occurs in the crown, which generates a necrotic orifice that is sometimes colonized by other secondary microorganisms. In the root system, there is darkening, reduction, damage, and orange-brown coloration in the vascular ducts [11]. Foa seems to be an aggressive pathogen to the different celery cultivars used in Costa Rica, which are mostly reported as resistant to *Fusarium* spp. (according to the commercial importer’s report).

Sequences of four races Epstein et al. [16] reported in the United States were compared to sequences from Costa Rican isolates using the molecular region of two genes, the EF-1α and the IGS rDNA. They found that races 1, 3, and 4 are similar in those genetic sequences but have significant differences in virulence that allow the pathogen to evolve into new races. Genetic variation on specific sites of the sequences from the isolates analyzed was observed along the population, and five specific single nucleotide mutations, which differentiate race 3 from races 1, 2, and 4 were present in all Costa Rican sequences. This allowed us to confirm the presence of race 3 in the country. Even though the populations from the different races are clonal and invariant [16], the Costa Rican population also shows variability within mutants in race 3. Additionally, we observed phenotypic variation in the behavior when presenting symptoms in celery plants.

Races 2 and 4 are reported as the most aggressive in California, US, with race 4 being highly virulent against all tested celery cultivars. Race 3 has been reported as presenting no risk to celery production in the world [16]. However, in Costa Rica, race 3 and other similar isolates, called mutants, are the ones causing great losses to celery producers. Henry et al. [13] reported that race 4 is closely related to, but distinct from, Foa race 3 and speculated that Foa race 4 arose from a Foa race 3-like strain, which may have arisen from one of the polyphyletic Foa race 1 strains.

High variability separates the closely related sequences to race 3, in three haplotypes; all of them are highly virulent and produce significant economic losses to farmers. They affect all cultivars of commercial celery imported into the country and are present in all sampled geographical locations. Zarcero, which has only grown celery in greenhouses, has a unique haplotype with low variation, probably due to low selection pressure since there is a lower use of fungicides. On the other hand, Cartago and San Jose, where celery crops grow in open fields, present a higher genetic variation along the areas. Finally, three haplotypes are reported in Cartago, which are present in all five cultivars used in Costa Rica and are reported to be tolerant to *Fusarium* spp., but it remains unknown whether these haplotypes are resistant to the race 3.

Costa Rica is not a celery seed producer. Seeds are imported from different countries around the world. Among these, the most used cultivar is David, which is imported from the Netherlands. Merengo and Triumph are also imported from the Netherlands. Gigante Verde is imported from the US and Isel is imported from the US, France, and India. There is no origin traceability of the seeds by the importing companies, and it is unknown from which material the plants were improved or from which importing markets. At the same time, Merengo and Isel showed the best results against race 3 closely related isolates. Gigante Verde is the most used cultivar in Zarcero, but it seems susceptible to the races isolated in this study. It has the advantage that most of the celery in Zarcero growing under greenhouse conditions improves the production of the crop. Cultivars in Cartago and Escazu are located mostly in open fields, which exposes them to fluctuations in tropical abiotic environmental conditions. 

The incursion of this pathogen in Costa Rica is likely due to the importation of seeds contaminated with Foa race 3, which, over the years, has allowed the pathogen to adapt to field conditions and become highly virulent. 

According to reports by Epstein et al. [17], the genetic improvement of celery cultivars has been carried out based on races 2 and 4, considered the most virulent races in the United States and other latitudes. After the establishment of race 2 in California, it was transported on infested seed to other states and countries such as Canada, Japan, and Argentina [17]. The low levels of resistance of seed cultivars imported to Costa Rica to race 3 may be due to the fact that most of the genetic improvement on celery cultivars has been conducted based on races 2 and 4 tolerance. Resistant cultivars such as Challenger have been used to adequately control Foa races 2 and 3 [13] but are susceptible to race 4 [18], and Tall Utah 52–70 R Improved is susceptible to Foa races 2 and 3; both could be tested in the country as alternatives to control the disease. Other commercial celery cultivars, such as Command, Green Bay, and Sabroso, have been the major tool for *Fusarium* yellows management since the early 2000s [13] but still are not present in Costa Rica.

Races 1 and 3, from *F. oxysporum* f. sp. *apii*, have been reported as the primary pathogen in celery and *Tithonia rotundifolia*, and as a secondary pathogen in a broad diversity of hosts, including cotton (*Gossypium barbadense* and *G. arboretum*), garden pea (*Pisum sativum*), sicklepod (*Cassia tora*) and eggplant (*Solanum melongena*) [16]. In recent studies, it has been demonstrated that Foa race 4 can cause disease in coriander (*Coriandrum sativum*) [17,26]. Additionally, it has been reported that coriander is a secondary host to Foa races 2 and 3, but these races are reported as less virulent than race 4 [13]. This poses threats to crop rotation using celery and coriander.

In countries such as the US, races 1 and 2 were controlled by the introduction of resistant cultivars, while race 4 required the development of more resistant germplasm [18]. Other control management, such as fumigation, use of fungicides, or rotation, has been reported as not effective for Foa control [16]. In Costa Rica or other seed-importing countries, it is important to understand the genetic diversity of the pathogen, which will allow the identification of resistance or tolerance germplasm (commercial seeds) to be used in the future. Greenhouse trials could be performed to measure the tolerance of commercial seeds to the haplotypes present in the country. The sequence of the whole genome will also help to elucidate the relation of the race presence to the reported in other studies [13,27].

Epstein and Kaur [18] recommended three issues for celery breeding: (1) Selection will require screening against the pathogen and the use of greenhouse trials with a consistent inoculum and a conducive temperature. According to Kaur et al. [5], when the temperature increases in the greenhouse, it could favor the development of some races more than others. (2) Since it is unknown how the pathogen can be removed from the soil and the inoculum density in soil correlates with the severity index [28], trials need to be performed in greenhouses or areas in which celery is not present, or other crops are grown. (3) Field tests should be undertaken in areas in which the pathogen (race 3) is already present.

Finally, it is extremely important to regulate and verify the health of the imported seeds to prevent the introduction of other races of Foa, which could greatly affect the celery crop and reach the country.

## 5. Conclusions

Multigene phylogenetic analysis was adequate to identify races and haplotypes on isolates from *Fusarium oxysporum* f. sp. *apii* (Foa) in Costa Rica. All isolates of Foa showed high virulence when used in commercial celery cultivars imported to the country, which are reported as resistant to *Fusarium* spp., probably race 2, but not resistant to race 3. Tolerance trials showed all isolates from Costa Rica are capable of affecting variables such as chlorosis, wilting, mortality, and fresh weight of the aerial part from celery plants. Cultivars such as Merengo and IseI showed the best results against Foa race 3. We conclude that celery producers in Costa Rica lack a commercial seed that could offer total resistance to the Foa race 3 present in the country. The information provided by this study could help establish trials for resistant and tolerant imported seeds using circulating Foa races to improve disease monitoring and the formulation of strategies for effective management, thus reducing yield losses of the celery crop. It is extremely important to establish country rules to prevent the introduction of other races of Foa in the seed material.

## Figures and Tables

**Table 1:** Analysis of variance using five response cultivars in an experiment with factorial arrangement for the tolerance of five celery cultivars and different isolates of *Fusarium oxysporum* f. sp. *apii* from celery plantations in Costa Rica.

	*p* Value				
Factor	Chlorosis ^1^	Wilting ^1^	Mortality ^1^	Plant Fresh Weight	Root Fresh Weight
Variety	<0.0001	<0.0001	<0.0001	<0.0001	0.4423
Treatment	<0.0001	<0.0001	<0.0001	<0.0001	0.4484
Variety*Treatment	<0.0001	<0.0001	<0.0001	<0.0001	0.4933

^1^ Before the analysis, an angular transformation of the data was performed.

## Data Availability

All data analyzed during this study are available from the corresponding author upon reasonable request.

## Supplementary Materials

The following supporting information can be downloaded at: https://www.mdpi.com/article/10.3390/microorganisms11112732/s1, Table S1: Primers used to sequence *Fusarium* isolates from Costa Rica; Figure S1. Five single nucleotides and one deletion that differentiate Foa race 3 from other races. Specific sites are present in all 58 sequences in the Costa Rican population.
