# Supplementary material for: Tolerance Evaluation of Celery Commercial Cultivars and Genetic Variability of Fusarium oxysporum f. sp. apii
Source: Microorganisms. 2023 Nov 9;11(11):2732. doi: 10.3390/microorganisms11112732 (PMC10673204; doi:10.3390/microorganisms11112732)
Supplement: Supplementary file 1 [file microorganisms-11-02732-s001.zip › microorganisms-2624973-SI.pdf]

Locus	Gene product	Size (bp)	Primer name	Sequence	Reference
TEF1	Translation elongation factor 1-α	700	EF1	ATGGGTAAGGA(A/G)GACAAGAC	[20]
			EF2	GGA(G/A)GTACCAGT(G/C)ATCATGTT	[20]
IGS	Intergenic spacer region of the rRNA gene	970	iNL11	AGGCTTCGGCTTAGCGTCTTAG	[21]
			FoIGS-R	GCCGACACCGCGCCTCTTAA	[16]

Table S1. Primers used to sequence *Fusarium* isolates from Costa Rica.

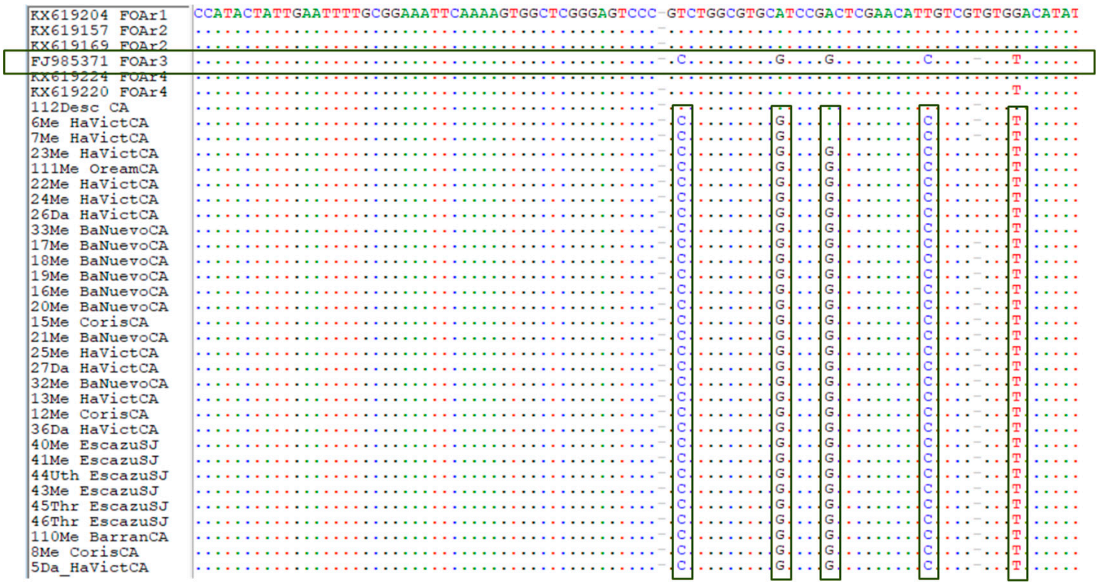

Figure S1. Five single nucleotides and one deletion that differentiate Foa race 3 from other races. Specific sites are present in all 58 sequences in the Costa Rican population.
